# Imaging disease activity of rheumatoid arthritis by macrophage targeting using second generation translocator protein positron emission tomography tracers

**DOI:** 10.1371/journal.pone.0222844

**Published:** 2019-09-25

**Authors:** S. T. G. Bruijnen, N. J. F. Verweij, Y. Y. J. Gent, M. C. Huisman, A. D. Windhorst, M. Kassiou, P. M. van de Ven, A. A. Lammertsma, O. S. Hoekstra, A. E. Voskuyl, C. J. van der Laken

**Affiliations:** 1 Amsterdam Rheumatology and Immunology Center (ARC), Amsterdam UMC| location VUmc, Amsterdam, the Netherlands; 2 Department of Radiology & Nuclear Medicine, Amsterdam UMC| location VUmc, Amsterdam, The Netherlands; 3 School of Chemistry, The University of Sydney, Sydney, Australia; 4 Department of Epidemiology and Biostatistics, Amsterdam UMC| location VUmc, Amsterdam, The Netherlands; Northwestern University Feinberg School of Medicine, UNITED STATES

## Abstract

**Background:**

Positron emission tomography (PET) imaging of macrophages using the translocator protein (TSPO) tracer *(R)*-[^11^C]PK11195 has shown the promise to image rheumatoid arthritis (RA). To further improve TSPO PET for RA imaging, second generation TSPO tracers [^11^C]DPA-713 and [^18^F]DPA-714 have recently been evaluated pre-clinically showing better imaging characteristics.

**Objective:**

A clinical proof of concept study to evaluate [^11^C]DPA-713 and [^18^F]DPA-714 to visualize arthritis in RA patients.

**Methods:**

RA patients (n = 13) with at least two active hand joints were included. PET/CT scans of the hands were obtained after injection of [^18^F]DPA-714, [^11^C]DPA-713 and/or *(R)*-[^11^C]PK11195 (max. 2 tracers pp). Standardized uptake values (SUVs) and target-to-background (T/B) ratios were determined. Imaging data of the 3 different tracers were compared by pooled post-hoc testing, and by a head to head comparison.

**Results:**

Clinically active arthritis was present in 110 hand joints (2–17 pp). Arthritic joints were visualized with both [^11^C]DPA-713 and [^18^F]DPA-714. Visual tracer uptake corresponded with clinical signs of arthritis in 80% of the joints. Mean absolute uptake in PET-positive joints was significantly higher for [^11^C]DPA-713 than for [^18^F]DPA-714, the latter being not significantly different from *(R)*-[^11^C]PK11195 uptake. Background uptake was lower for both DPA tracers compared with that of *(R)*-[^11^C]PK11195. Higher absolute uptake and lower background resulted in two-fold higher T/B ratios for [^11^C]DPA-713.

**Conclusions:**

[^11^C]DPA-713 and [^18^F]DPA-714 visualize arthritic joints in active RA patients and most optimal arthritis imaging results were obtained for [^11^C]DPA-713. Second generation TSPO macrophage PET provides new opportunities for both early diagnosis and therapy monitoring of RA.

## Introduction

International guidelines for rheumatoid arthritis (RA) stress the importance of starting effective treatment as early as possible [1]. It appears that early treat-to-target therapy results in delay or complete termination of progressive joint damage and in reduced disability [2, 3]. Sensitive and quantitative imaging techniques could add valuable information on (changes in) disease activity on top of clinical evaluation.

Positron emission tomography (PET) is a nuclear imaging technique with high sensitivity and potentially high specificity depending on the tracer being used for specific targeting of molecular sites of interest [4]. PET provides quantitative molecular data making it particularly interesting for early diagnosis and therapy monitoring. A potential target to assess RA disease activity is the macrophage, as macrophages infiltrate in synovium right from the early development of RA and they remain a relevant biomarker during treatment [5, 6]. PK11195 (1-[2-chlorophenyl]-N-methyl-N-[1-methyl-propyl]-3-isoquinoline carboxamide) (*(R)*-[^11^C]PK11195) binds to the upregulated translocator protein (TSPO) in activated macrophages (validated by histological examination of synovial RA tissue in relation to PET) and is able to visualize synovitis in both established and pre-RA patients [7, 8], the latter allowing early diagnostics of RA. Moreover, using this tracer, it was possible to predict flares in RA patients in clinical remission [9, 10]. However, the relatively high background uptake of *(R)*-[^11^C]PK11195 in peri-articular tissues hampered detection of more subtle synovitis.

Recently two new high affinity TSPO ligands were developed, DPA-714 (*N*,*N*-Diethyl-2-(2-[4-(2-fluoroethoxy)-phenyl]-5,7-dimethyl-pyrazolo[1,5-a]pyrimidin-3-yl)-acetamide) [11] and DPA-713 (*N*,*N*-diethyl-2-[2-(4-methoxyphenyl)-5,7-dimethylpyrazolo[1,5-a]pyrimidin-3-yl]acetamide) [12]. Although *(R)*-[^11^C]PK11195, [^18^F]DPA-714 and [^11^C]DPA-713 all bind to TSPO in the nanomolar range, *in vitro* studies show an increased binding of [^18^F]DPA-714 and [^11^C]DPA-713 to TSPO compared with *(R)*-[^11^C]PK11195 [13–15]. In addition, they have shown more favourable target-to-background ratios than *(R)*-[^11^C]PK11195 in arthritic joints of a rat arthritis model [16]. Recently, another second generation TSPO tracer [^11^C]-PBR28 showed higher uptake in joints of RA patients than in healthy joints and PBR28–specific binding in synovial tissue was approximately 10-fold higher in RA patients than in healthy controls [17].

The purpose of the present study was to explore whether TSPO PET can be further optimized for arthritis imaging by investigation of the second generation TSPO PET tracers [^18^F]DPA-714 and [^11^C]DPA-713 for the first time in clinically active RA patients in a clinical proof of concept setting.

## Material and methods

### Patients

Thirteen RA patients (ACR 2010) [18] were included between November 2013 and July 2016. Eligible patients (>18 years) had at least two swollen joints in hands/wrists. Stable treatment with Disease Modifying Anti-Rheumatic drugs (DMARDs), oral corticosteroids (maximum 10 mg/day), stable doses of non-steroidal anti-inflammatory drugs (NSAIDs, ≥1 month) and/or biologicals (≥3 months) were permitted. Patients were excluded if they had been treated with investigational drugs within the previous three months, or if they were pregnant or breastfeeding.

The study protocol was approved by the VUmc Medical Ethics Review Committee. All patients gave written informed consent before participation in the study.

### Study design

[^11^C]DPA-713, [^18^F]DPA-714 or *(R)*-[^11^C]PK11195 PET/CT scans of both hands/wrists were performed for all RA patients. Due to radiation limits, a maximum of 2 consecutive tracers were investigated in the same patient. Therefore, tracers could partly be compared head to head by injecting two different tracers per patient (see data in S1 Appendix). All patients started with a C-11 labeled tracer followed by an F-18 labeled tracer, with a minimal interval period of 3 hours between the two scans (i.e. ~9 times the half-life of carbon-11).

### Synthesis of *(R)*-[^11^C]PK11195, [^18^F]DPA-714 and [^11^C]DPA-713

Radiopharmaceuticals were synthesized according to Good Manufacturing Practice (GMP) in a facility with a manufacturing license at the VU University campus (Amsterdam, The Netherlands).

*(R)*-[^11^C]PK11195 has previously been used for clinical studies at the VU University Medical Center and was produced accordingly [8]. In addition, [^18^F]DPA-714 has previously been administered to healthy volunteers and patients with neurological disorders. The DPA-714 tracer was synthesized by fluorination of a tosyl precursor as previously described [11]. [^11^C]DPA-713 was synthesized according to the procedures of Thominiaux et al. by methylation with [^11^C]methyl-triflate of its demethylated precursor [12].

### (R)-[^11^C]PK11195, [^18^F]DPA-714 and [^11^C]DPA-713 PET/CT scanning

Scans were obtained using Gemini TF or Ingenuity TF PET/CT scanners (Philips Medical Systems, Cleveland, Ohio, USA). No fasting was needed and patients received an infusion needle (Venflon®) before the scan for blood withdrawal to assess TSPO polymorphism rs6971 (see below). Subsequently, patients were injected with a mean ± standard deviation (SD) of 440±6 MBq *(R)*-[^11^C]PK11195 (n = 3) and/or 176±7 MBq (n = 8) [^18^F]DPA-714 and/or 356±36 MBq [^11^C]DPA-713 (n = 10), respectively. As TSPO tracers may easily stick to syringes and tubing, the injection syringe was flushed with 20 mL of NaCl 0.9%, and residual activity was measured.

*(R)*-[^11^C]PK11195 scans of the wrists/hands (consisting of two field of views (FOVs)) were started 20 minutes after intravenous injection as described before [8]. In addition, hands were placed in a special vacuum pouch for stabilization. As the optimum scan intervals for the new TSPO tracers were not known, [^18^F]DPA-714 and [^11^C]DPA-713 scans were started 10 minutes after injection and a total of four emission scans (mean 9 mins per scan) were obtained in the time interval 10–50 minutes p.i. (data in S2 Appendix). Scan time was adjusted for the half-life of the applied isotope.

PET scans were preceded by a low dose 35mAs CT scan for attenuation correction and localization purposes. The maximum total scan time was approximately 40 minutes per patient per tracer. All scans were reconstructed according to previously used protocols [19].

### Image analysis

Scans (~20 min p.i) were first visually analyzed to determine which joints were PET positive for consecutive quantitative analysis. PET/CT images of the hands were evaluated for PET-positive joints in wrists, metacarpophalangeal (MCP) joints and proximal interphalangeal (PIP) joints (n = 22 per patient). Reading was carried out in random order of tracers per patient by two readers blinded for clinical findings (SB, CL). A joint was assessed as PET positive when tracer uptake was visually higher than extra-articular tissue (dichotomously).

For quantitative comparison of tracer uptake, volumes of interests (VOIs) were drawn on PET images using in-house developed data analysis software with the covering low-dose CT as anatomical reference. Each emission scan was analyzed separately and VOIs were drawn on top of the visually determined PET-positive joints using automatic, threshold based, isocontours. At a lesion level, the uptake in extra-articular metacarpal bone per patient was used as lowest threshold.

Standardized uptake values (SUVs) were calculated by dividing the PET tracer tissue concentration in each VOI by the injected radioactivity and normalizing it to body weight. Tracer uptake in targets is presented as SUVpeak, which is defined as the highest average uptake within a sphere of 1.2 mL within the VOI [20, 21]. In addition, standardized spherical VOIs were drawn in a non-affected metacarpal (MC) bone (preferably the second MC bone) to determine background uptake in bone and to calculate target-to-background (T/B) ratios.

Analysis was performed using the mean SUV and T/B ratios per joint defined as the average of the four consecutive measurements of [^18^F]DPA-714 and [^11^C]DPA-713, based on a low coefficient of variation (CV) (median 0.07; IQR 0.05–0.13) and a high correlation (r = >0.97) between the SUV values of PET-positive joints as derived from the four consecutive emission scans.

### Determination of TSPO polymorphism rs6971

The homozygous TSPO polymorphism rs6971 is known to affect the binding affinity of second generation TSPO tracers [22]. For correct interpretation of data the genetic TSPO status of patients was determined.

The “QI Amp DNA Blood Mini Kit (Qiagen #51104, Hilden, Duitsland)” was used for rapid purification of genomic deoxyribonucleic acid (DNA), essentially as described before [23]. In short, blood was collected in EDTA blood collection tubes. Genomic DNA was isolated from 200 μL whole blood using the QIAamp DNA Blood Mini Kit according to the manufacturer’s instructions. Genomic DNA was eluted in 200 μL buffer AE after which purity and concentration was assessed using the NanoDrop-1000 Spectrophotometer (Isogen, Utrecht, The Netherlands). A260/280 ratios were typical> 1.8. 10 ng of gDNA was mixed with TaqMan Genotyping Master Mix (Applied Biosystems P/N 4371353, California, United States) and Taqman SNP Genotyping Assay (Applied Biosystems P/N 4351379, Assay ID C_2512465_20, SNP id rs6971) and nuclease-free water in a total volume of 20 μL. QPCR was performed in MicroAmp Optical 96-well plates (Applied Biosystems) on a StepOnePlus Real-Time PCR system (Applied Biosystems). Homozygous TSPO polymorphism patients were classified A/A whereas the other patients were either heterogeneous (A/G) or lacked polymorphism (G/G).

### Statistical analysis

Results of the visual interpretation of PET/CT images were analyzed in a descriptive, dichotomous (a joint scored either PET positive or negative) manner.

The quantitative analyses of mean absolute and relative levels of uptake in PET-positive joints (SUV and T/B ratios) were determined for each tracer. Subsequently, pooled data per tracer were compared between the three tracers using a mixed model with a random effect for joint-subject combination and a fixed effect for tracer. The random effect was included to account for each subject receiving two of the 3 tracers. In case a significant overall difference between the three tracers was found, post-hoc tests were performed to compare levels of uptake between each pair of tracers. A Bonferroni correction was used to account for multiple testing in the post-hoc analyses. In addition, pooled results in the mixed model were corrected for joint size (measured ordinally with a semi-quantitative categorization, i.e. wrist = 1, MCP = 2, PIP = 3) and genetic polymorphism status.

In addition, next to pooled tracer data analysis, head to head comparisons were performed, comparing mean SUV and T/B ratios between the two scans in subsets of patients who obtained a specific pair of scans. These analyses are presented separately. Comparisons between tracers were performed using the non-parametric Mann-Whitney.

The relation between PET data and clinical parameters were determined by Spearman’s rank correlation. Categorical variables are summarized by frequencies and percentages. Continuous variables are summarized using mean ± SD and 95% confidence interval (95% CI) or as median and interquartile range [IQR] in case of a non-normal distribution.

A p-value <0.05 was regarded as statistically significant and statistical analyses were performed using SPSS version 22.0.0 for Windows (SPSS, Chicago, IL, USA).

## Results

### Clinical data

Baseline demographics of the 13 RA patients are summarized in Table 1. Out of 286 evaluated hand/wrist joints, 101 were clinically tender and/or swollen (35%) (2–17 joints per patient). The distribution of clinically active joints was 15% (15/101) wrists, 50% (50/101) metacarpophalangeal (MCP) joints and 35% (36/101) proximal phalangeal (PIP) joints, respectively.

**Table 1:** Baseline patient demographics, clinical and functional characteristics.

N = 13	
Females, number (%)	7(54%)
Age, years	59±14
Length, cm	175±8
Weight, kg	81 [75–87]
IgM RF^¥^ positivity, number (%)	8 (61.5)
Anti-CCP^¥^ positivity, number (%)	8 (61.5)
Disease duration, years	3.0 [1.3–15.0]
DAS28	5.4 [4.0–5.6]
Swollen joint count	7.0 [3.0–12.5]
Tender joint count	8.0 [2.5–14.5]
VAS	55.0 [40.0–64.0]
CRP, mg/mL*	13.0 [3.3–15.0]
ESR, mm/h	23.0 [13.0–38.0]
NSAID use, number (%)	5 (38%)
DMARD use, number (%)	12 (92%)
Prednisone, number (%)	7 (54%)
Dosage in mg/day	7.5 [2.5–10.0]

Values are presented as absolute number (%), mean±SD or median[IQR].

IgM RF, Rheumatoid Factor; anti-CCP, Anti-cyclic citrullinated peptide; DAS28, disease activity score of 28 joints; VAS, visual analogue scale for pain; CRP, C-reactive protein; ESR, erythrocyte sedimentation rate; DMARD, Disease-modifying antirheumatic drugs.

* CRP lower detection limit is 2.5 mg/mL.

_¥_ Lower limit for IgM RF = 5.0 IU/ml and for a-CCP = 10,0 U/ml, respectively.

### PET data in relation to clinical disease activity

**Fig 1:**
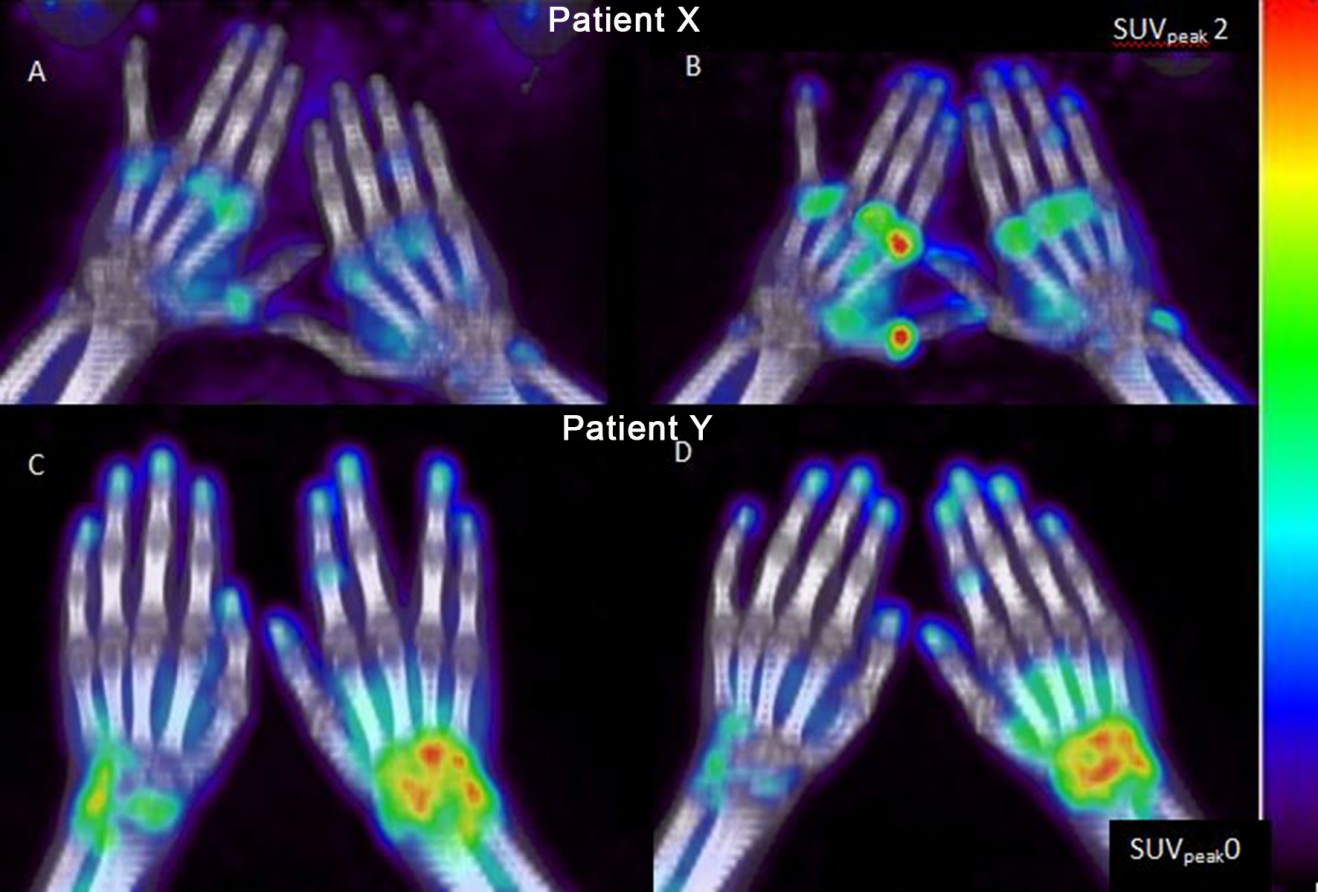
PET/CT Maximum Intensity Pictures show accumulation of the three TSPO tracers. (A) *(R)*-[^11^C]PK11195 and (B) [^18^F]DPA-714 images within one patient (patient X), and (C) [^18^F]DPA-714 and (D) [^11^C]DPA-713 images within another patient (patient Y).

[^11^C]DPA-713 and [^18^F]DPA-714 accumulated in up to 7 joints per patient, which could be identified on PET/CT (range 2–7) (Fig 1).

At a group level, visual (dichotomous) interpretation of the PET/CT scans revealed that the distribution and the number of PET-positive joints were identical for both DPA tracers and similar to that of the reference tracer *(R)*-[^11^C]PK11195(e.g. Fig 1). A total of 49 visual PET-positive joints were observed. Eighty percent (39/49) of PET-positive joints also showed clinical signs of arthritis. PET-positive joint distribution consisted of 26% (13/49) wrists, 37% (18/49) MCP joints and 37% (18/49) PIP joints, respectively. In 74% (175/237) of PET-negative joints, no clinical symptoms of arthritis were present. Lack of a PET-positive signal in clinically inflamed joints (i.e. tender and/or swollen) was more often found in the small MCP (70%) and PIP (61%) joints as compared with the larger wrist joints (33%).

At a patient level, the total number of visually assessed PET-positive joints (regardless of the tracer) and the mean SUV values of all PET-positive joints per patient did not correlate with clinical parameters (i.e. DAS28 and TJC, SJC), disease duration andESR. Only the mean SUV per patient of [^11^C]DPA-713 showed a very strong correlation with C-reactive protein (CRP) (r = 0.8; p = 0.009), although this was not found for the other TSPO tracers.

### Comparison of pooled quantitative PET data of tracers

**Fig 2:**
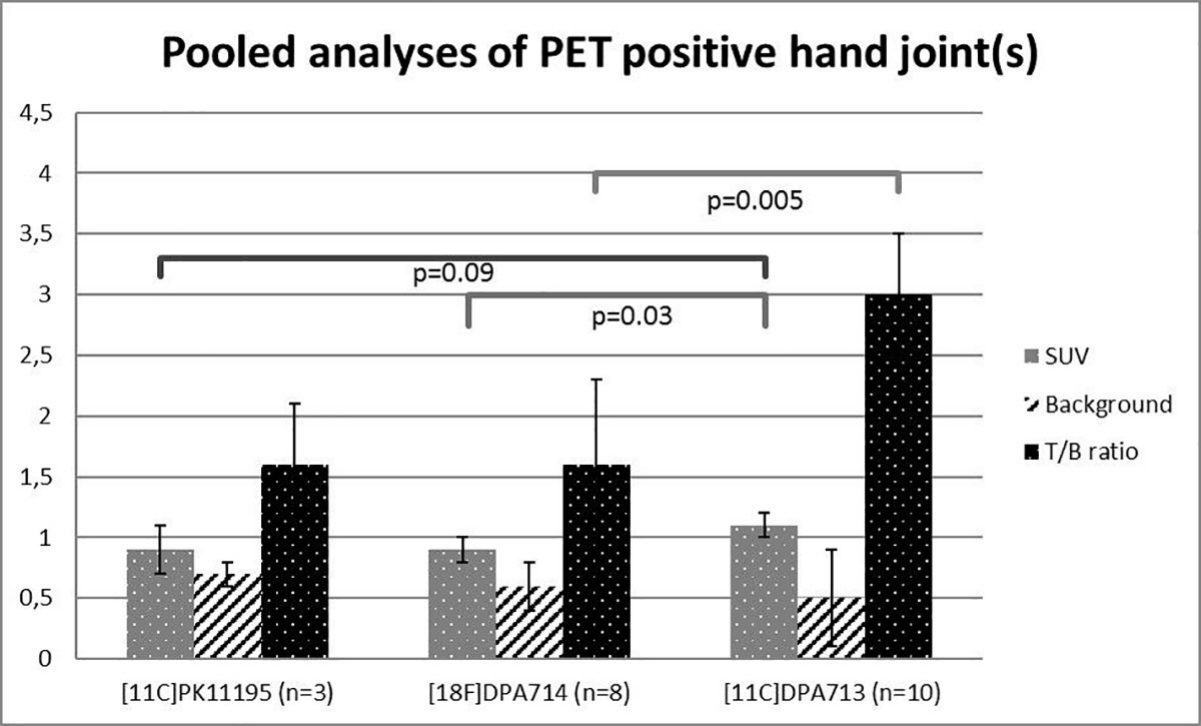
Mean (± SD) SUV (left; grey), background SUV (middle; white striped) and T/B ratios (right; black bars) of the pooled analyses per tracer in hand joints among RA patients.

Mean SUVs for [^18^F]DPA-714, [^11^C]DPA-713 and *(R)*-[^11^C]PK11195 in visually identified PET-positive joints are presented in Fig 2. The mean SUV of [^11^C]DPA-713 was significantly higher than that of [^18^F]DPA-714 (p = 0.03) and tended to be higher than that of *(R)*-[^11^C]PK11195 (p = 0.09, Fig 2). The mean SUV values of PET-positive joints were not different for [^18^F]DPA-714 and *(R)*-[^11^C]PK11195 (p = 1.0).

There was a trend for lower background uptake in metacarpal bone for [^11^C]DPA-713 and, to a lesser extent, [^18^F]DPA-714 than for *(R)*-[^11^C]PK11195 (Fig 2). The higher absolute uptake in arthritic joints and lower background of [^11^C]DPA-713 resulted in an almost twofold higher T/B ratio than that of [^18^F]DPA-714 and *(R)*-[^11^C]PK11195. Despite a slightly lower background uptake of [^18^F]DPA-714 as compared with *(R)*-[^11^C]PK11195, the T/B ratios of these two tracers were not significantly different (Fig 2). The overall p-value for comparing T/B ratios of the three tracers was p = 0.007, the Bonferroni correct p-value for post-hoc comparison for tracers showed only significant differences between [^11^C]DPA-713 and [^18^F]DPA-714 (p = 0.005).

Quantitative analysis at a joint level revealed that joints, which were both PET positive and clinically active (swelling and/or tenderness), had significantly higher [^11^C]DPA-713 and [^18^F]DPA-714 SUV values and T/B ratios than PET-positive joints that were clinically not active ([^11^C]DPA-713: SUV 1.1±0.7 vs 0.6±0.4, p<0.001; T/B 3.4±3.2 vs 1.3±0.6, p<0.001; [^18^F]DPA-714: SUV 1.1±0.6 vs 0.7±0.5, p = 0.02, T/B 2.0±1.0 vs 1.3±0.5, p = 0.04). For *(R)*-[^11^C]PK11195 no significant differences were found between clinically active and non-active joints that were PET positive (p = 0.7).

After correction for the TSPO tracers (fixed effect) used in the mixed model, mean SUV and T/B ratios were significantly higher in larger joints (i.e. wrists versus PIP joints, p<0.001). This difference remained after correcting for semi-quantitative joint size (i.e. wrist = 1 to PIP = 3) (p = 0.049).

### Head to head comparison of tracers

The tracers that were injected in the same patients could also be compared in a head-to-head setting. Results of these analyses were comparable with those of the pooled tracer data analyses. There was a significantly higher SUV for [^11^C]DPA-713 than for [^18^F]DPA-714 (1.0±0.8 vs 0.8±0.6 in 71 joints; p<0.001)), but not for [^18^F]DPA-714 versus *(R)*-[^11^C]PK11195 (1.3±0.3 vs 1.3±0.3 in 8 joints;(p = 0.9). A direct head to head comparison between *(R)*-[^11^C]PK11195 and [^11^C]DPA-713 was not available in this study (see data in S1 Appendix).

### TSPO polymorphism

Homozygous rs6971 polymorphism (A/A) was found in 2 out of 13 patients which is thought to be related to a “low binding” status for the TSPO tracers. The other patients (n = 11) were either heterogeneous (A/G; n = 5) or lacked polymorphism (G/G; n = 6). Correction of the pooled quantitative PET measures for polymorphism status did not alter the results regarding the significant differences between the tracers, neither for SUV nor for T/B ratios.

## Discussion

This clinical proof of concept study is the first study evaluating the feasibility to visualize arthritic joints of RA patients by PET/CT and macrophage targeting using the second generation TSPO tracers, [^11^C]DPA-713 and [^18^F]DPA-714. PET scans with both tracers clearly depicted arthritis. PET data of both DPA tracers confirmed previous findings, obtained with the first generation TSPO tracer *(R)*-[^11^C]PK11195, that TSPO is an appropriate target for non-invasive imaging of macrophages in RA, reflecting disease activity. Although differences between TSPO tracers were relatively small and obtained in small groups, the data show that arthritis imaging by TSPO targeting on macrophages can be improved further by using a second generation TSPO tracer, most pronounced for [^11^C]DPA-713 with a reduction in background uptake and an increase in target uptake.

Although most PET findings corresponded with clinical assessments, there were also some small discrepancies. For instance, in 20% of PET-positive joints, clinical examination did not reveal arthritis activity, which may point at imaging of subclinical inflammation. This is in line with previous *(R)*-[^11^C]PK11195 findings in both pre-RA and established RA in clinical remission, where PET positive, but clinically negative joints were associated with development of clinical disease activity later in time [7, 10]. Conversely, 26% of PET-negative joints showed signs of clinical inflammation. This discrepancy may be due to several factors. First, clinical identification of pain and/or swelling of a joint may rely on extinguished inflammation or at osteoarthritis without the presence of actual inflammation, which may explain a negative finding on PET. Second, small patient movement could have hampered correct reconstruction of the PET signal. Although an attempt was made to prevent motion of the hands, small movements could not always be avoided, for example, due to breathing with the hands positioned on the abdomen. In addition, the limited spatial resolution of PET (4-6mm) may have affected the sensitivity of PET to depict inflammatory activity in the smaller hand joints. The discordancy between PET and clinical findings was indeed mainly found in MCP and PIP joints. Future studies may benefit from dedicated high-resolution scanners.

By investigating two tracers within a single patient on the same day, it was possible to perform head to head comparisons in addition to the pooled tracer data analysis. Within one patient, there was no competition between the two tracers for binding due to very small (tracer) amounts of injected tracers and an excess of binding sites present in inflamed joints [24]. In addition, the time interval between injections of the tracers was chosen such that the second tracer was injected after the first tracer had decayed. The comparisons between tracers were statistically corrected for comparison within the same patients. Results were similar to those from the pooled analysis of all tracer comparisons, reinforcing the conclusions.

In this study, the TSPO polymorphism status did not affect the comparisons between the TSPO tracers for arthritis imaging. Previous data obtained in human brain, indicated that all second-generation TSPO tracers, including the two DPA tracers of the present study, showed reduced binding affinity in patients homozygous for this polymorphism compared to the wild type [22]. This was not found for the first-generation TSPO tracer *(R)*-[^11^C]PK11195. In the present study, both DPA tracers visualized arthritic joints independent of polymorphism status, suggesting a lack of effect of TSPO polymorphism status on arthritis targeting which would be an advantage for clinical implementation.

The group sizes were relatively small, in particular the subgroup of patients that were injected with *(R)*-[^11^C]PK11195. Most likely, this contributed to the lack of reaching statistical differences in absolute joint uptake (SUV) between [^11^C]DPA-713 and *(R)*-[^11^C]PK11195. Interestingly, statistical differences were found between [^11^C]DPA-713 and [^18^F]DPA-714, which showed similar levels of uptake as *(R)*-[^11^C]PK11195. The small group sizes probably also played a role in finding no statistically significant association between [^18^F]DPA-714 and CRP, while this was found for [^11^C]DPA-713 and CRP. As absolute joint uptake was lower for [^18^F]DPA-714 than for [^11^C]DPA-713, it would require a larger subgroup size to find a statistically significant association with CRP.

For both DPA tracers, data were analyzed using uptake values that were averaged over the four consecutive emission scans. There is a risk of perfusion effects in the earliest of the four emission scans (~<25mins). Indeed, on average, there was a slight increase in SUV over time between emission scans 1 and 4 (data not shown). Nevertheless, the average of scans 1–4 strongly correlated with the average of scans 3 and 4 alone. Therefore, to increase statistical accuracy, the average of scans 1–4 was used in the present analysis.

High sensitivity and quantitative accuracy make PET a promising tool for clinical applications within rheumatology, such as early diagnostics and therapy monitoring [25]. Most optimal arthritis imaging results in this study were obtained for [^11^C]DPA-713. The use of radioisotype carbon-11 has advantages for monitoring studies (repetitive scanning), as the half-life of carbon-11 is shorter, resulting in a lower radiation burden allowing for more repeat scans in the same subject. Although arthritis imaging results of [^18^F]DPA-714 were only marginally better than of *(R)*-[^11^C]PK11195, the use of a F-18 radio-isotype may be of interest for clinical practice since the longer half-life of [^18^F]DPA-714 allows for central (commercial) production with distribution to various sites.

## Conclusions

The current data confirm the potential of TSPO PET for non-invasive imaging of RA disease activity by targeting previously histologically validated TSPO on (activated) macrophages in synovial tissue. Imaging characteristics of arthritis can be further improved by usingthe second generation TSPO tracer [^11^C]DPA-713. Non-invasive identification of arthritis activity by TSPO PET may have value for clinical applications of early diagnosis and therapy monitoring of RA, which should be further investigated in future studies.

## Supporting information

S1 Appendix Study and analysis design. (DOCX)

S2 Appendix Scanprotocol. (DOCX)

S3 Appendix Radiolabeling of PK11195, DPA-713, DPA-714. (DOCX)
