# Supplementary material for: Imaging disease activity of rheumatoid arthritis by macrophage targeting using second generation translocator protein positron emission tomography tracers
Source: PLoS One. 2019 Sep 25;14(9):e0222844. doi: 10.1371/journal.pone.0222844 (PMC6760780; doi:10.1371/journal.pone.0222844)
Supplement: S1 Appendix — (DOCX) [file pone.0222844.s001.docx]

**Appendix A: Study and analysis design.**

| [^11^C]-(R)-PK11195  Patient # | [^18^F]DPA-714  Patient # | [^11^C]DPA-713  Patient # |
| --- | --- | --- |
| 1 | 1 |  |
| 2 | 2 |  |
| 3 | 3 |  |
|  | 4 | 4 |
|  | 5 | 5 |
|  | 6 | 6 |
|  | 7 | 7 |
|  | 8 | 8 |
|  |  | 9 |
|  |  | 10 |
|  |  | 11 |
|  |  | 12 |
|  |  | 13 |
| Head to head #1-3 | Head to head # 1-3 |  |
|  | Head to head # 4-8 | Head to head # 4-8 |
|  |  |  |
| Pooled #1-3 versus | Pooled #1-8 versus | Pooled # 4-13 |

Pooled results were compared between the three tracers using a mixed model with a random effect for joint-subject combination and a fixed effect for tracer. The random effect was included to account for each subject receiving two of the three tracers.
