# Supplementary material for: Imaging disease activity of rheumatoid arthritis by macrophage targeting using second generation translocator protein positron emission tomography tracers
Source: PLoS One. 2019 Sep 25;14(9):e0222844. doi: 10.1371/journal.pone.0222844 (PMC6760780; doi:10.1371/journal.pone.0222844)
Supplement: S2 Appendix — (DOCX) [file pone.0222844.s002.docx]

**Appendix B: Scanprotocol.**

|  | [^11^C]-(R)-PK11195  5min/bed position |  |  | [^18^F]DPA-714  4 minutes/bed position | [^11^C]DPA-713  3-5-5-7 minutes/bed position |
| --- | --- | --- | --- | --- | --- |
|  |  |  | Scan 1  (2 bedpositions) | ±10 – 18  min p.i. | ±10 – 16  min p.i. |
| Scan 1  (2 bedpositions) | ±20-30  min p.i. |  | Scan 2  (2 bedpositions) | ±20 – 28  min p.i. | ± 18 – 28  min p.i. |
|  |  |  | Scan 3  (2 bedpositions) | ±30 – 38  min p.i. | ± 30 – 40  min. pi. |
|  |  |  | Scan 4  (2 bedpositions) | ±40 – 48  min p.i. | ± 42 – 56  min p.i. |

**Minutes/bed position: total duration in minutes of one static (overlapping) bed position, with each scan consisting of 2 bed positions; min p.i.: minutes post injection of the tracer. Visual analysis was performed on the scans obtained at 20-30 minutes post injection (dichotomous scoring, grey bar).**
