# Supplementary material for: Imaging disease activity of rheumatoid arthritis by macrophage targeting using second generation translocator protein positron emission tomography tracers
Source: PLoS One. 2019 Sep 25;14(9):e0222844. doi: 10.1371/journal.pone.0222844 (PMC6760780; doi:10.1371/journal.pone.0222844)
Supplement: S3 Appendix — (DOCX) [file pone.0222844.s003.docx]

***S3 Appendix: Radiolabeling of PK11195, DPA-713, DPA-714.***

All radiopharmaceuticals are synthesized according to GMP compliant procedures.

[^11^C]-*(R)-*PK11195: ^11^CO_2_ is produced by irradiation of ^14^N/O_2_ (95.5/0.5%) with 18 MeV protons. ^11^CO_2_ is harvested by condensation at -150 ^0^C. Subsequently, ^11^CO_2_  is reacted with lithiumaluminiumhybride in tetrahydrofuran. ^11^CH_4_ is formed. The tetrahydrofuran is evaporated in vacuo. Hydrogeniodide is added yielding [^11^C]methyliodide. Thereafter, [^11^C]methyliodide is distiled into a mixture of desmethyl-(*R*)-PK11195, potassiumhydroxide and dimethylsulfoxide. After completion of the reaction, (*R*)-[^11^C]PK11195 is purified by HPLC. (*R*)-[^11^C]PK11195 is extracted from the HPLC elute by solid phase extraction and formulated with a mixture of sterile ethanol, a solution of sodiumdihydrogenphosphate and saline. The solution of (*R*)-[^11^C]PK11195 is filtered by inline filtration. After quality control analyses and approval of the hospital pharmacist the solution is ready to inject.

The (*R*)[^11^C]PK11195 injection solution has a specific activity of > 2295 MBq/μmol. The radiochemical purity is > 98%. The solution contains organic solvents acetonitril, tetrahydrofuran and dimethylsulfoxide, each < 50 ppm, endotoxins < 8.75 iU/ml (Vmax-20 ml). The solution is colorless and sterile. The pH is 5-8.

[^11^C]DPA-713 was synthesized by methylation with [^11^C]methyl-triflate of its demethylated precursor (donated by Prof Michael Kassiou, University of Sydney, Australia). The precursor (1 mg) was dissolved in 300 µL acetonitrile and 2.5 µL 0.5M NaOH was added. To this solution [^11^C]methyl-triflate was added at -10°C. After reaction for 5 min at 100°C, the reaction mixture was cooled down to 20°C and diluted with 0.7 mL of water for injection. This total solution was injected onto a Reprospher 100 C18-DE, 5 µm HPLC column which was eluted with a 40/60 mixture of Acetonitrile/0.1% DIPEA. The product, [^11^C]DPA-713, eluted at 7~8 min. This fraction was collected in 60 ml water for injection. The total solution was passed over a preconditioned (10 mL of sterile ethanol 96% and subsequently 10 mL of water for injection) Waters SEPPAK tC 18. The cartridge was washed with 20 ml of water for injection and subsequently the product was eluted from the cartridge with 1.0 ml of sterile ethanol (96%) and 14 ml of a sterile and pyrogen free sodiumphosphate solution (7.09 mM, pH 5.4) in saline. This final mixture was passed over a Millex GV 0.22 µm filter, yielding a sterile, isotonic and pyrogen free solution of 1-5 GBq of [^11^C]DPA-713. The product was analyzed with HPLC on a Xterra C-18 MS 5 µm column which was eluted with 60/40 mixture of Ammonium dihydrogenphosphate pH 2.5 / Acetonitril.
 **[^11^C]DPA-713 specifications**

| Parameter | Specification | Release specification |
| --- | --- | --- |
| Appearance | Clear and colourless liquid | Yes |
| pH | 5 – 8 | Yes |
| HPLC (System suitability test):  - System equilibrated (SST)  - ΔRt duplos reference (SST)  - Blank formulation solution (SST)  HPLC Analysis:  - Radiochemical purity  - Radiochemical identity  - Chemical purity | Equilibration chromatogram stable  < 1 min (Rt 6-8 min)  Identical to master chromatogram  ≥ 98 %  ΔRt of product and reference < 1 minute  Besides injection and carrier peak, no other UV peaks than present in blank | Yes  Yes  Yes  Yes  Yes  Yes |
| Specific Activity (ART) | ≥ 18,5 GBq/µmol (>500 Ci/mmol) | Yes |
| Integrity filter | Luchtweerstand steriel filter ≥ 2 bar | Yes |
| Aceton | ≤ 50 ppm | No |
| Acetonitril | ≤ 50 ppm | No |
| Tetrahydrofuraan | ≤ 50 ppm | No |
| Dimethylsulfoxide | ≤ 50 ppm | No |
| Ethanol concentration | 5 – 8 % | No |
| Half life | 18 - 22 min | No |
| Sterility | Sterile | No |
| Bacterial endotoxine content | Sample value ≤ 2,5 EU/ml  (Vmax = 70 ml) | No |

[^18^F]DPA714 was synthesized by fluorination of a tosyl precursor (donated by Prof Michael Kassiou, University of Sydney, Australia). After standard work-up of the aqueous [^18^F]fluoride, the precursor (1.5-2.5 mg) in 0.5 mL acetonitrile was added. After reaction for 10 min at 105°C, the reaction mixture was diluted with 3 mL of water. This total solution was injected onto a Waters XTerra RP18 (5 µm, 10x250 mm) HPLC column which was eluted with a 65:35 mixture of 0.1 M ammonium acetate and acetonitrile. The product, [^18^F]DPA-714, eluted at 20 min. This fraction was collected in 40 mL water for injections. The total solution was passed over a preconditioned (5 mL of sterile ethanol 96% and subsequently 10 mL of water for injections) Waters Sep-Pak C18 Plus cartridge. The cartridge was washed with 20 mL of water for injection and subsequently the product was eluted from the cartridge with 0.9 mL of sterile ethanol (96%) and chased with 9 mL of a sterile and pyrogen-free 0.9% sodium chloride solution. This final mixture was passed over a Millex GV 0.22 µm filter, yielding a sterile, isotonic and pyrogen-free solution of 4159 GBq (SD 2282) of [^18^F]DPA-714. The product was analyzed with HPLC on a Chromolith RP-18e (100x4.6 mm) column which was eluted with 70:30 mixture of a sodium phosphate buffer pH 3.5 and acetonitrile.

**[^18^F]DPA-714 specifications**

| Parameter | Specification | Release Specification |
| --- | --- | --- |
| Appearance | Clear and colourless liquid | Yes |
| pH | 5.0 – 8.0 | Yes |
| HPLC (System suitability test):  - System equilibrated (SST)  - ΔRt duplos reference (SST)  - Blank formulation solution (SST)  HPLC Analysis:  - Radiochemical purity  - Radiochemical identity  - Chemical purity | Equilibration chromatogram stable  < 1 min (Rt 4 - 6 min)  Identical to master chromatogram  ≥ 95 %  ΔRt of product and reference <1 minute  Besides injection and carrier peaks, no other UV peaks than present in blank | Yes  Yes  Yes  Yes  Yes  Yes |
| Bubble point 0.22 µm filter | > 2 bar | Yes |
| Specific Activity at end of synthesis | ≥ 28 GBq/µmol | Yes |
| Radioactive concentration | > 50 MBq/mL | No |
| Chemical impurities (total) | < 0.09 µg/mL | Yes |
| Kryptofix concentration | ≤ 50 µg/mL | Yes |
| Acetone | ≤ 5000 ppm | No |
| Acetonitrile | ≤ 410 ppm | No |
| Ethanol concentration | 7.5 – 11.5 % | No |
| Sterility | Sterile | No |
| Bacterial endotoxine content | ≤ 17.5 EU/mL | Yes |
| Half-life | 105 – 115 minutes | No |
| Radionuclidic purity | ≥ 99.9% ^18^F | No |
